# Evolutionary Divergence of Duplicated *Hsf* Genes in *Populus*

**DOI:** 10.3390/cells8050438

**Published:** 2019-05-10

**Authors:** Bobin Liu, Jianjun Hu, Jin Zhang

**Affiliations:** 1 College of Forestry, Fujian Colleges and Universities Engineering Research Institute of Conservation & Utilization of Natural Bioresources, Fujian Agriculture and Forestry University, Fuzhou 350002, China; liubobin@fafu.edu.cn; 2 State Key Laboratory of Tree Genetics and Breeding, Key Laboratory of Tree Breeding and Cultivation of the National Forestry and Grassland Administration, Research Institute of Forestry, Chinese Academy of Forestry, Beijing 100091, China; hujj@caf.ac.cn; 3 Biosciences Division, Oak Ridge National Laboratory, Oak Ridge, TN 37831, USA

**Keywords:** *Populus*, heat shock transcription factors, gene duplication, gene expression, alternative splicing, single nucleotide polymorphism, protein structure, co-expression network

## Abstract

Heat shock transcription factors (Hsfs), which function as the activator of heat shock proteins (Hsps), play multiple roles in response to environmental stress and the development of plants. The Hsf family had experienced gene expansion via whole-genome duplication from a single cell algae to higher plants. However, how the Hsf gene family went through evolutionary divergence after genome duplication is unknown. As a model wood species, *Populus trichocarpa* is widely distributed in North America with various ecological and climatic environments. In this study, we used *P. trichocarpa* as materials and identified the expression divergence of the *PtHsf* gene family in developmental processes, such as dormant bud formation and opening, catkins development, and in response to environments. Through the co-expression network, we further discovered the divergent co-expressed genes that related to the functional divergence of *PtHsfs*. Then, we studied the alternative splicing events, single nucleotide polymorphism distribution and tertiary structures of members of the *PtHsf* gene family. In addition to expression divergence, we uncovered the evolutionary divergence in the protein level which may be important to new function formations and for survival in changing environments. This study comprehensively analyzed the evolutionary divergence of a member of the *PtHsf* gene family after genome duplication, paving the way for further gene function analysis and genetic engineering.

## 1. Introduction

Heat shock proteins (Hsps), which function as molecular chaperones to maintain proteostasis, are critical for almost all organisms in response to environmental stresses, such as heat, cold, drought, salt, biotic stresses and so on [1,2,3,4]. Heat shock transcription factors (Hsfs) play essential roles from fungi to plants and animals in response to environmental stress to induce the transcription of Hsps via binding to the heat shock elements (HSEs) that are located in their upstream promoter regions [1,2,5]. Except for the stress response, Hsf is also involved in developmental processes in animals and plants [1,2], such as oogenesis and larvae development in *Drosophila Melanogaster* [6] and seed development in *Arabidopsis* [7]. Therefore, Hsf is a fundamental transcription factor (TF) family in organism development and surviving in a fluctuating environment, especially for plants because of their sessile lifestyle. 

Hsf proteins are highly conserved from fungi to animals. The first Hsf was identified from *D. melanogaster* cells via the DNA-protein interaction method [8]. A typical Hsf protein contains three conserved domains that are the DNA-binding domain (DBD), the oligomerization domain including heptad repeat A (HR-A) or HR-B, and the carboxy-terminal HR-C [2]. The DBD is the most conserved domain which can bind to HSEs of the target genes, and also can interact with other TFs to regulate the transaction activity of Hsf [2]. The HR-A and HR-B contributed Hsf trimerization by forming a coiled-coil structure, but HR-C mediated the suppression of Hsf trimerization [2]. The human genome encodes six Hsfs which exhibit diversity neofunctionalization [9]. In plants, the *Hsf* gene family was expanded due to the whole-genome duplication events, for instance, there are 21 Hsf genes in *Arabidopsis* [10], 27 Hsf genes in *Salix suchowensis* [11] and 28–32 Hsf genes in the *Populus* species [12,13]. According to the amino acid length from DBD to HR-A/B and the length between HR-A and HR-B, plant Hsfs were classed into three main subclasses (A, B, and C) [10]. Like in animals, the function of Hsfs in plants also shows diversity. Many studies reported that Hsfs in plants have critical roles in the response and adaption to various environmental dynamic changes and in developmental processes [1,14], which means the expanded Hsf genes have undergone subfunctionalization or neofunctionalization processes during plant evolution. However, how the expanded genes from the genome duplication acquire new functions in the plant evolution history is unclear.

The *Populus* genus is wildly distributed in the northern hemisphere with various ecological conditions [15]. It has important commercial value in the word because of its rapid growth and abundant biomass [15,16]. Now numerous poplar species and hybrid elite were planted globally for producing wood, fiber, biofuel, and so on [17,18]. In addition, poplar has a critical ecology benefit, such as soil and water conservation, anti-desertion, carbon fixation, etc. [19]. *Populus trichocarpa* became a wood model species after whole-genome sequence [20], genetic transformation system establishment and abundant genetic resource collection [21,22]. *P. trichocarpa* is natively distributed in western North American from southern Alaska to California with a variety of ecological and climate environments which means *P. trichocarpa* possess a high plasticity of adaptive phenotypic variation in growth, vegetative phenology and physiological traits [23,24]. Because of the important roles of Hsf in environment adaption, we previously analyzed the expression patterns of *Hsf* genes in different tissues and under different stress conditions in *P. trichocarpa* [12], but how the evolutionary events affect functional divergence of the *Hsf* gene family in *Populus* is still unknown. 

In this study, we further analyzed the *PtHsf* genes’ expression patterns during developmental processes based on the latest gene expression atlas database, which included dormant bud formation and open, catkins development, leaf expansion, and response to nitrogen. We found that the expression patterns were divergent in the *PtHsf* gene family which was consistent with the potential function. We further studied the alternative splicing, SNP distribution and 3D structures of members of the PtHsf gene family, uncovered protein level evolutionary divergence that may be related to the new function formation and mechanisms of plants surviving in different environments. 

## 2. Materials and Methods

### 2.1. Characteristics of *Populus Hsf* Genes

To identify *Populus Hsf* genes, the published *Arabidopsis* Hsf protein sequences [25] were searched against the latest *P. trichocarpa* genome (release V3.1) through tBLASTn. All homologous protein sequences of the Hsf candidates were accepted if they were satisfied with the expectation value (*E*) < 1E-40. To further confirm the evolution history, protein sequences of *Gnetum montanum*, *Taxus baccata*, *Ginkgo biloba* and *Picea abies* were downloaded from Plaza (https://bioinformatics.psb.ugent.be//plaza/versions/gymno-laza/download#collapse_func_annot), others were downloaded from phytozome v12 (https://phytozome.jgi.doe.gov/pz/portal.html). A Hidden Markov Models (HMM) profile of Hsf (PF00047) downloaded from the Pfam database (http://pfam.xfam.org/) was queried against the protomes via hmmersearch to identify the Hsf gene family as described previously [26]. All proteins with an expectation value (*E*) < 1 × e^−10^ and harbored the Hsf domain confirmed in the Conserved Domain Database (https://www.ncbi.nlm.nih.gov/cdd/) were used for further analysis. The phylogenetic tree was constructed using IQ-TREE 1.6.9 [27] with the JTT + F + I + G4 model that was the best-fit model selected by ModelFinder [28] and ultrafast bootstrap (1000 replicates) for each alignment [29]. The interactive Tree Of Life [30] was used to display and edit the phylogenetic tree.

### 2.2. Promoter Analysis

The promoter sequences (2 kb upstream of translation initiation site) of *PtHsfs* were downloaded from Phytozome (https://phytozome.jgi.doe.gov/pz/portal.html#). The similarity of the promoters between the *PtHsf* paralogous pair was analyzed using the default parameter of PlantPAN 3.0 [31].

### 2.3. Gene Expression and Co-Expression Network

Expression data of *PtHsfs* in various tissues were obtained from the *Populus* Gene Atlas Study (https://phytozome.jgi.doe.gov/phytomine/aspect.do?name=Expression), which including 24 tissues/treatments: (1) Pre-dormant bud I, (2) Pre-dormant bud II, (3) Early dormant bud, (4) Late dormant bud, (5) Fully open bud (6) GW9592.ZK 10 male early, (7) GW9840.ZE 30 male early, (8) GW9911.ZK 51 male mid (9) BESC423.ZL 7 female early, (10) BESC842.ZI 22 female late, (11) BESC443.ZG 43 female receptive (12) Leaf immature standard, (13) Leaf young standard, (14) Leaf first fully expanded standard, (15) Root standard, (16) Root tip standard, (17) Root ammonia, (18) Root nitrate, (19) Root urea, (20) Stem node standard, (21) Stem inode standard, (22) Stem ammonia, (23) Stem nitrate, (24) Stem urea. The data used for the heatmap was log 2 transformed. Co-expression relationships of *PtHsfs* were downloaded from Phytozome (https://phytozome.jgi.doe.gov/pz/portal.html#). The genes with a Pearson Correlation Coefficients (PCC) greater than or equal to 0.85 and a *P* value < 0.05 were used for the co-expression network construction by Cytoscape [32].

### 2.4. Splice Variants of *PtHsfs*

The splice variants of *PtHsfs* were obtained from the latest annotation release of *P. trichocarpa* (V3.1) from Phytozome (https://phytozome.jgi.doe.gov/pz/portal.html#) with the default parameter.

### 2.5. Natural Variation in *PtHsfs*

The single nucleotide polymorphisms (SNPs) in the *PtHsf* genes were obtained from Phytozome, which was based on the whole genome re-sequencing data of 549 *P. trichocarpa* natural individuals in North America [22]. Based on the SNPs location and their consequence, the SNPs were classified into UTR 5 prime, UTR 3 deleted, UTR 3 prime, intron, start gained, start lost, stop gained, stop lost, frame shift, codon insertion, codon deletion, codon change plus codon deletion, codon change plus codon insertion, splice site donor, splice site acceptor, synonymous stop, synonymous coding and non-synonymous coding.

### 2.6. Protein Structural Modeling

The 3D structures of PtHsfs were built using the Iterative Threading ASSEmbly Refinement (I-TASSER, v.5.1, Lansing, MI, USA) protein structure modeling toolkit [33] with the default parameter.

## 3. Results

### 3.1. Genome-Wide Duplication of *Hsf* Genes in Populus

**Figure 1:**
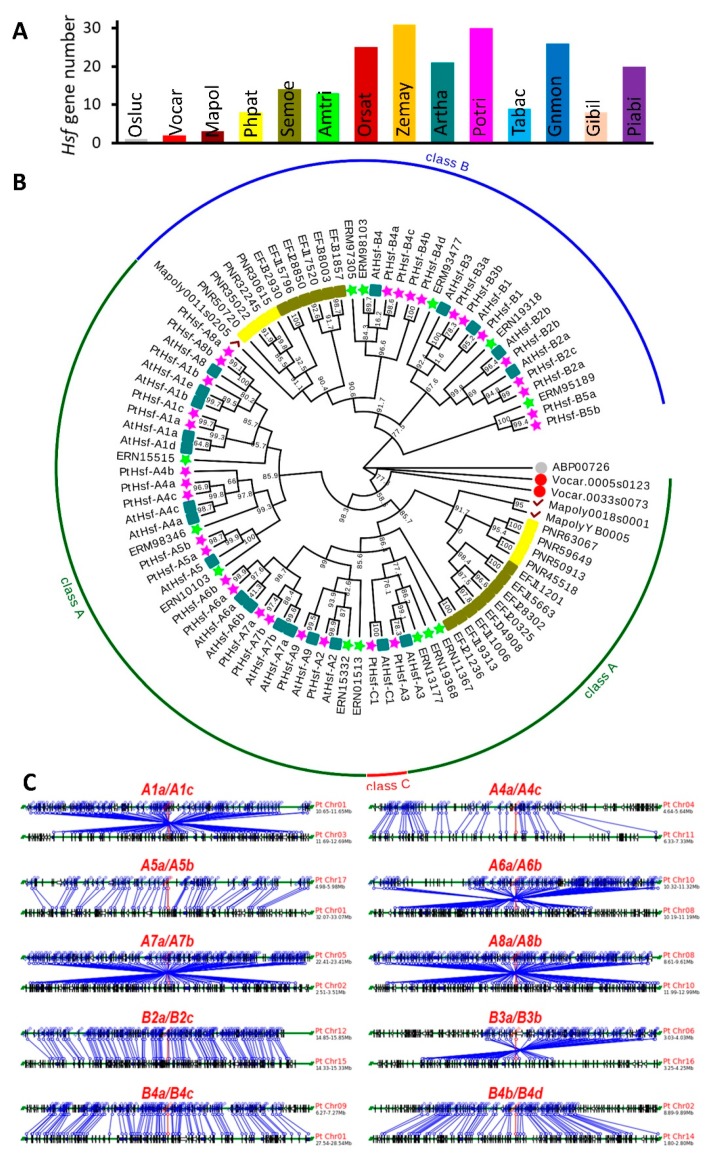
The *Hsf* gene family underwent gene expansion in evolution history. (**A**) The gene number of *Hsf* genes in different plants. Note that the *Hsf* gene number was multiplied from algae to land plants. *Ostreococcus lucimarinus* (*Osluc*), *Volvox carteri* (*Vocar*), *Marchantia polymorpha* (*Mapol*), *Physcomitrella patens* (*Phpat*), *Selaginella moellendorffii* (*Semoe*), *Amborella trichopoda* (*Amtri*), *Arabidopsis thaliana* (*Artha*), *Populus trichocarpa* (*Potri*), *Oryza sativa* (*Orsat*), *Zea mays* (*Zemay*), *Gnetum montanum* (*Gnmon*), *Taxus baccata* (*Tabac*), *Ginkgo biloba* (*Gibil*) and *Picea abies* (*Piabi*). (**B**) Evolutionary relationship of the *Hsfs* from *Osluc*, *Vocar*, *Mapol*, *Phpat*, *Semoe*, *Amtri*, *Artha* and *Potri*. Note that every subfamily *Hsf* genes in *Arabidopsis* and poplar were expanded from *Amborella trichopoda*, the oldest angiosperm. (**C**) Syntenic relationships of duplicated genes in the *PtHsf* family.

To study the *Hsf* gene number variation during the evolution from algae to land vascular plants, we identified *Hsf* genes from 14 plants that include plant species from lower algae to higher vascular plants (Figure 1A). Only 1 and 2 *Hsf* genes were in the *Ostreococcus lucimarinus* and *Volvox carteri* genome, respectively (Figure 1A). The *Hsf* gene number increased in the earlier land plants, having 3 and 7 *Hsfs* in *Marchantia polymorpha* and *Physcomitrella patens*, respectively. When plants evolved to vascular plants and higher seed plants, the *Hsf* genes number were doubled (Figure 1A). This means the *Hsf* genes were multiplied on the way to terrestrial plants and seed plants. In order to study the evolutionary relationships of *Hsf* genes from aquatic to higher plant, we construct a phylogenetic tree using the full length of the Hsf protein sequences from the 8 plant species including *Ostreococcus lucimarinus*, *Volvox carteri*, *Marchantia polymorpha*, *Physcomitrella patens*, *Selaginella moellendorffii*, *Amborella trichopoda*, *Arabidopsis thaliana* and *Populus trichocarpa* (Figure 1B). The *Hsf* gene originated in *Ostreococcus lucimarinus*, a unicell algae and divided into 3 main subclasses that were subclass A, B and C in the evolution history (Figure 1B). Both subclass A and B contained a branch consisting of *Hsf* genes from *Marchantia polymorpha*, *Physcomitrella patens*, *Selaginella moellendorffii*, and *Hsf* showed expansion in the *Physcomitrella patens* and *Selaginella moellendorffii* genome. Subclass A and B were further divided into different subfamilies (A1–A9 in the subclass A, B1–B5 in the subclass) in *Amborella trichopoda* that was considered as the basal angiosperms [34]. *Hsf* genes from higher plants exhibited expansion in every subfamily when evolved from *Amborella trichopoda*. This result suggested that there were *Hsf* gene expansion processes when the higher plant evolved from aquatic life. 

To investigate how the *PtHsf* gene family expanded during whole-genome duplication, we found that 23 out of 28 *PtHsf* were located on duplicated fragments and ten paralogous pairs were identified via phylogenetic analysis [12]. Here we compared the syntenic relationships of genes nearby the duplicated *PtHsfs*. As shown in Figure 1C, 1 Mb chromosome blocks containing duplicated *PtHsfs* were used to compare the syntenic relationship. Among the ten *PtHsf* paralogous pairs, four *PtHsf* pairs (*A6a/A6b*, *A7a/A7b*, *A8a/A8b*, *B4a/B4c*, and *B4b/B4d*) were located in high density duplicated blocks, while two *PtHsf* pairs (*A4a/A4c* and *A5a/A5b*) were located in relative low-density duplicated blocks. This result indicates that the *PtHsf* gene family underwent expansion and evolution via whole genome duplication. 

### 3.2. Expression Similarity and Divergence of *PtHsf* Genes

**Figure 2:**
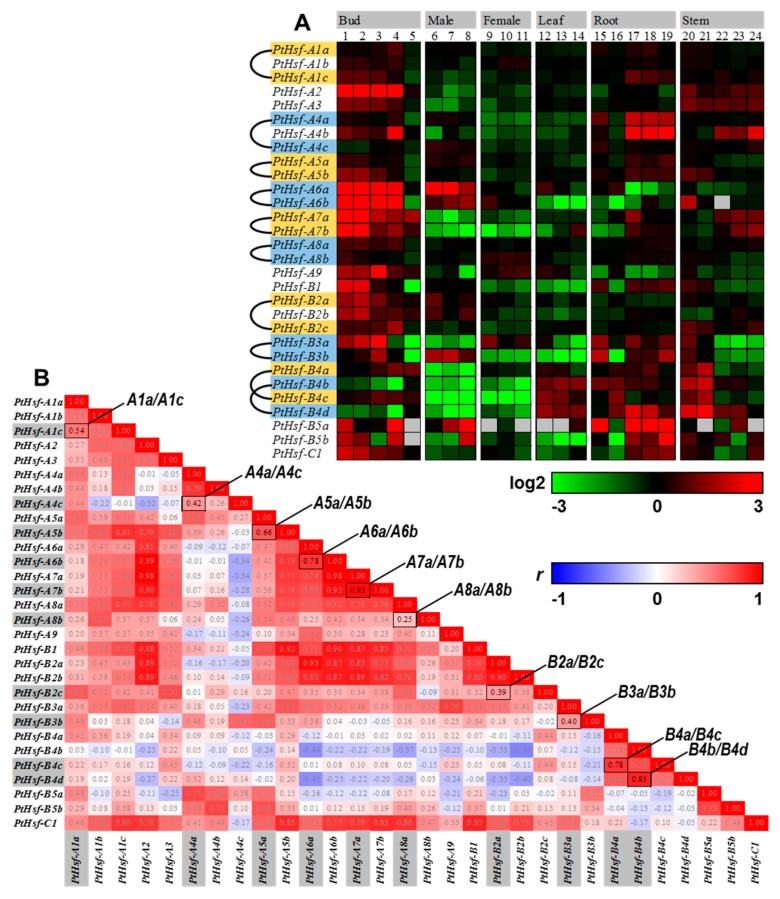
The expression patterns of *PtHsf* and the pairwise correlation co-efficiency. (**A**) Expression patterns of *PtHsfs* across various tissues. (1) Pre-dormant bud I, (2) Pre-dormant bud II, (3) Early dormant bud, (4) Late dormant bud, (5) Fully open bud (6) GW9592.ZK 10 male early, (7) GW9840.ZE 30 male early, (8) GW9911.ZK 51 male mid (9) BESC423.ZL 7 female early, (10) BESC842.ZI 22 female late, (11) BESC443.ZG 43 female receptive (12) Leaf immature standard, (13) Leaf young standard, (14) Leaf first fully expanded standard, (15) Root standard, (16) Root tip standard, (17) Root ammonia, (18) Root nitrate, (19) Root urea, (20) Stem node standard, (21) Stem inode standard, (22) Stem ammonia, (23) Stem nitrate, (24) Stem urea. (**B**) Pairwise comparison of Pearson correlation co-efficiency (PCC, *r*).

To explore whether the gene function is divergent with the expansion of the *PtHsf* gene family, we analyzed the expression patterns of *PtHsfs*. The gene expression level in different tissues or under different growth conditions reflects its potential function in the organism. Based on the *Populus* gene atlas database, we compared the expression patterns of *PtHsfs* in different developmental processes such as bud set and bud flush, male/female catkin development, leaf expansion, root/stem response to different nitrogen nutrition (Figure 2). In dormant buds, most of the *PtHsf* genes were highly expressed except *PtHsf-A4c*, *-B4b* and *-B4d* (Figure 2A), which means most of the *PtHsfs* genes were involved in dormant bud formation. This might be a strategy to protect the meristem to survive in the winter [35,36]. In fully opened buds, most of *PtHsfs* were down-regulated obviously except *PtHsf-A7a*, *-B2b*, *-B4a, -B4b, -B4c and -B4d* (Figure 2A). The down-regulation of *PtHsfs* in fully opened buds might be caused by seasonal variation. From dominant bud formation to release processes, we found that almost all *PtHsfs* play important roles in response to dynamic environmental change (Figure 2A). 

In male catkin development, three *PtHsfs* (*-A6a*, *-A6b* and *-B3b*) were consistently highly expressed, five *PtHsf* (*-B2a, -B2b, -B2c*, *-B5a* and *-B5b*) showed dynamic changes of transcription abundance, but other *PtHsfs* showed low expression levels or without obvious expression changes (Figure 2A). Noticeably, the expression patterns of three paralogous pairs (*A1a/A1c*, *B2a/B2c* and *B3a/B3b*) showed significant differences in male catkin development. In female catkin development, *PtHsf-A8b*, *-A9* and *-B3a* showed a relative high transcription level, the other *PtHsfs* were down-regulated (Figure 2A). Two paralogous pairs (*A6a/A6b* and *B3a/B3b*) showed different expression patterns in female catkin. Interestingly, the paralogous pair *B3a/B3b* showed a divergent expression pattern in male/female catkins, implying that *PtHsf-B3a* and *PtHsf-B3b* might play alternative dominant roles in female and male catkin development, respectively (Figure 2A). During the leaf expansion process, eight *PtHsfs* (*-A6a*, *-A6b*, *-A7b*, *-A9*, *-B2a*, *-B3a*, *-B4a* and *-B5a*) showed dynamic changes in ‘immature’—‘young’—‘fully expand’ transition, three *PtHsfs* in the B4 subfamily (*-B4b*, *-B4c* and *-B4d*) were maintained in the higher expression level (Figure 2A). Among the paralogous pairs, two pairs (*A6a/A6b* and *B3a/B3c*) showed significant divergence (Figure 2A). In addition, we analyzed the nitrogen response of *PtHsfs* in the root and stem, the paralogous pair *B3a/B3b* were induced by nitrogen treatments in the root but were repressed in the stem (Figure 2A). These results indicate that most paralogous pairs in the *PtHsf* gene family showed divergent gene expression patterns either in various developmental stages or under different nutrition conditions. 

In order to better understand the expression divergence of *PtHsfs*, especially between paralogous pairs, the Pearson correlation coefficient (PCC, *r*) was employed to establish relationships among the *PtHsf* gene family (Figure 2B). Noticeably, only two of the ten *PtHsf* paralogous pairs showed a strong positive correlation (*A7a/A7b* and *B4b/B4d*) with *r* > 0.8 (Figure 2B). This further suggests that the duplicated *Hsf* genes underwent subfunctionalization or neofunctionalization during the evolution.

### 3.3. Promoter Similarity between the *PtHsf* Paralogous Pairs

**Figure 3:**
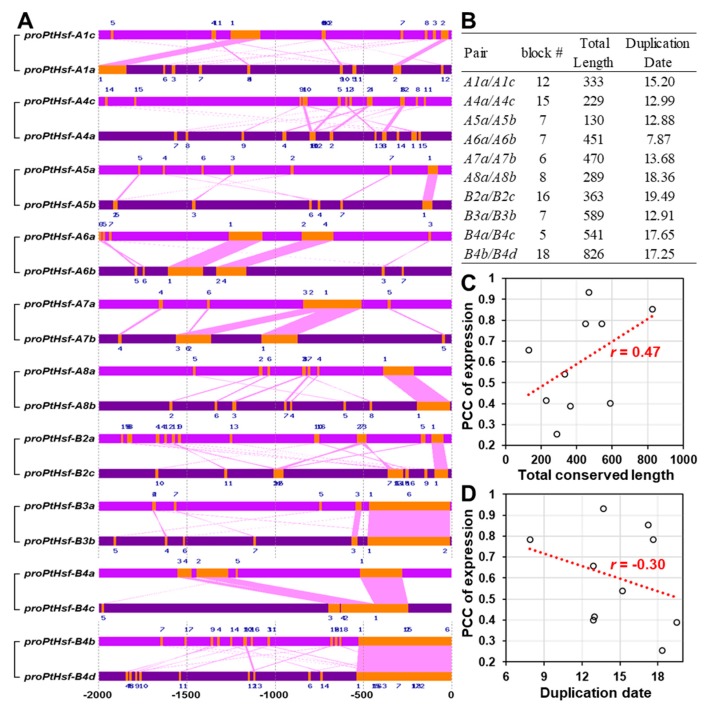
The promoter similarity between 10 gene pairs in the *PtHsf* family. (**A**) Conserved blocks located in the promoter region of *PtHsf* gene pairs. (**B**) Conserved block number and total conserved length between the PtHsf pairs’ promoter. (**C**) The correlation coefficiency between the total conserved length and the PCC of the expression (Figure 2) of *PtHsf* pairs. (**D**) The correlation coefficiency between the paralogous pair duplication date and the PCC of expression.

The gene expression pattern depends on the *cis*-acting elements that are located in the gene promoter region. We then analyzed the promoter similarity between *PtHsf* paralogous pairs. Among the ten *PtHsf* paralogous pairs, *B4b/B4d* showed the highest sequence similarity in the 2 kb promoter region (Figure 3A,B). To test if the conserved promoter sequence is associated with the gene expression similarity or duplicated data, we performed a correlation analysis. Noticeably, the gene expression correlation coefficient between paralogous pairs was positively correlated with the length of the conserved promoter regions; but was negatively correlated with the duplication date (Figure 3C,D). This indicates that the promoter similarity directly affects the gene expression similarity and the differences of promoter similarity might be caused by the evolution process.

### 3.4. Co-Expression Network of *PtHsfs*

**Figure 4:**
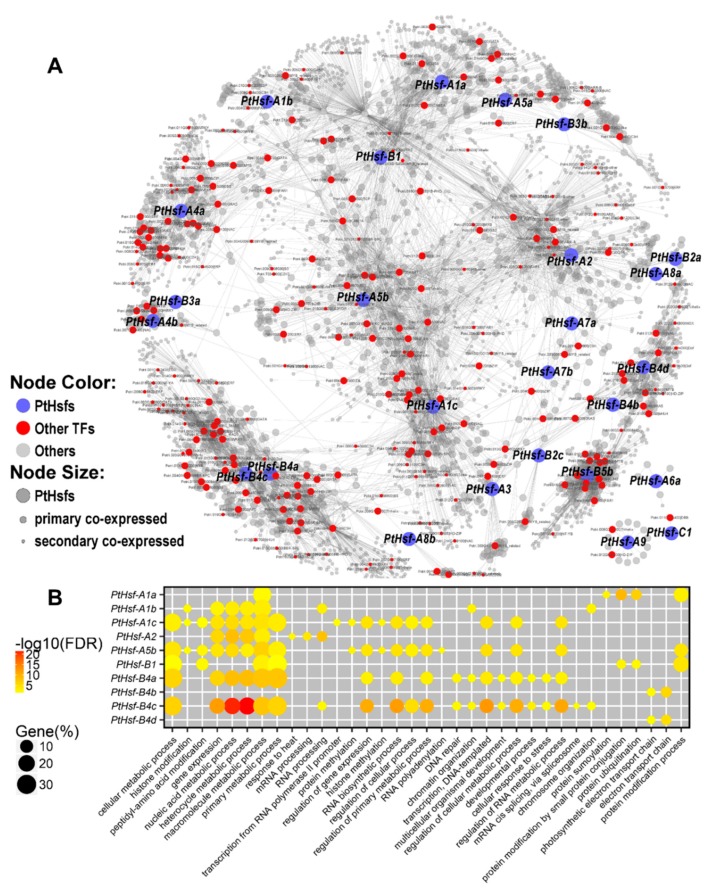
The co-expression network of *PtHsfs*. (**A**) Co-expression network of *PtHsfs*. Blue nodes indicate PtHsfs and red nodes indicate other transcription factors (TFs). (**B**) Gene ontology (GO) enrichment analysis co-expression sub-networks of ten *PtHsfs* genes. Yellow to red represents -log10 transformed false discovery rate and node size indicates the percentage of GO enriched genes. GO enrichment was shown in Table S1.

Generally, functional-associated genes share similar expression patterns since they are regulated by the same upstream regulators or they are in the same regulatory pathway. Co-expression network provides clues to discover genes’ potential function and their evolutionary divergence [37]. Based on the genome-wide *Populus* expression atlas database, we constructed a *PtHsfs* co-expression network (Figure 4A). A total of 4468 genes were co-expressed with the *PtHsf* genes except for four members (*PtHsf-A4c*, *PtHsf-A6b*, *PtHsf-B2b* and *PtHsf-B5a*), and the co-expressed gene number varied from 2 to 626 for different *PtHsf* genes (Table 1 and Figure 4A). *PtHsf-A9*, *PtHsf-A6a* and *PtHsf-C1* were co-expressed with 15, 6 and 2 genes and formed three independent sub-networks, respectively (Figure 4A). The remained 23 *PtHsfs* co-expressed with 4445 genes and consisted of a complex co-expression network (Figure 4A). In the co-expression network, *PtHsf-A1c*, *PtHsf-A2*, *PtHsf-A1b*, *PtHsf-A1a*, *PtHsf-A5a*, *PtHsf-B3b* and *PtHsf-A5b* were located in a largest sub-network with their co-expressed genes, but *PtHsf-B4a/4c*, *PtHsf-B4b/4d*, *PtHsf-A3a/4b*, *PtHsf-A4a* and *PtHsf-B5b* were located in an independent sub-network, respectively (Figure 4A); the other 11 *PtHsfs* were scattered in the co-expression network (Figure 4A). Only 2 out of 10 paralogous pairs were in the same sub-network (Figure 4A). This result indicates that although most of *PtHsf* genes were in a co-expression network, their co-expression relationships were relatively independent. This implies that the *PtHsf* genes generated by whole-genome duplication events underwent functional divergence. 

In order to further reveal the potential functions of the *PtHsf* genes, we performed multiple functional analyses including gene ontology (GO) enrichment, protein domain enrichment, pathway enrichment and enriched TF analysis based on the co-expressed genes (Table 1, Table S1 and Figure 4B). At the threshold of the false discovery rate corrected *P*-value < 0.05, only 10 out of 23 *PtHsf* sub-networks were enriched with a different number of GO terms which varied from 6 to 59 (Figure 4B and Table S1). The partial of the GO term is consistent with the potential function based on the expression pattern. The enriched GO term of the *PtHsf-A1b* sub-network represented in the function of “chromosome organization”, “chromatin organization”, “chromatin modification”, “covalent chromatin modification” and “histone modification” (Table S1), might be involved in the meiotic cell cycle in catkins development [38]. *PtHsfB4s* were highly expressed in the leaf expansion process (Figure 2A). This process included cell enlarge, cell wall thicken and photosynthesis enhancement [39]. We found that the *PtHsf-B4a/B4c* sub-network was enriched in GO terms of “cellular aromatic compound metabolic process”, “cellular macromolecule metabolic process”, “macromolecule metabolic process” and “primary metabolic process” (Table S1), implying that *PtHsf-B4a/B4c* were involved in cell construction. In contrast, the *PtHsf-B4b/B4d* sub-network was enriched in “electron transport chain”, “respiratory electron transport chain”, “photosynthetic electron transport chain”, and “photosynthetic electron transport in photosystem II” (Table S1), indicating that *PtHsf-B4b/B4d* play roles in photosynthesis. This result suggested that the two paralogous pairs (*PtHsf-B4a/B4c* and *PtHsf-B4b/B4d*) in the subfamily B4 play totally different roles in leaf development. In response to different nitrogen treatments, “nitrogen compound metabolic and biosynthetic processes” related GO terms were enriched in the sub-networks of *PtHsf-A1b*, *PtHsf-A1c*, *PtHsf-A2*, *PtHsf-A5b*, *PtHsf-B4a* and *PtHsf-B4c*, which were dynamic responses to nitrogen treatments (Figure 2A, Table S1). In addition, the sub-networks of *PtHsf-A1b*, *PtHsf-A1c*, *PtHsf-A2*, *PtHsf-A5b*, *PtHsf-B1*, *PtHsf-B4a*, *PtHsf-B4c* were mainly enriched in the “primary, macromolecule, heterocycle and cellular metabolic process” and “gene expression” (Figure 4B), which might be caused by a function redundant or functional diversity.

In the protein domain enrichment analysis, we found that the sub-networks of 15 *PtHsfs* were enriched in 1–41 protein domains, of which the Hsp and Hsf protein domains were enriched in the sub-networks of *PtHsf-A2*, *PtHsf-A7a* and *PtHsf-B2a* (Table S1), which are consistent with their dominant roles in the heat shock response [12]. Different domain types were enriched in the sub-networks of *PtHsf* genes, except *PtHsf-B4b*/*B4d* (Table 1 and Table S1). Noticeably, the distinct domain enrichment divergences were exhibited among the sub-networks of *PtHsf-A1b, PtHsf-A1c, PtHsf-A2, PtHsf-A5b, PtHsf-B1, PtHsf-B4a* and *PtHsf-B4c*, which showed conserved GO enrichment (Table 1 and Table S1). We further found that the co-expressed TFs in the sub-networks of *PtHsfs* also displayed divergence in the *PtHsf* gene family. For example, the sub-networks of *PtHsf-B4b* and *PtHsf-B4d* were enriched in similar GO terms and functional domains, but their co-expressed TFs were significantly different. Only two TFs in the HD-ZIP family (Potri.001G372300 (homolog of *Arabidopsis HB14*, *PHB*) and Potri.001G188800 (homolog of *Arabidopsis HB15*, *CNA*)) were co-expressed with both *PtHsf-B4b* and *PtHsf-B4d*. For sub-network-specific TFs, one *bHLH* and two *GRAS* TFs were specifically co-expressed with *PtHsf-B4b*, whereas one *NAC*, one *bHLH*, one *WOX* and two *TCP* TFs were specifically co-expressed with *PtHsf-B4d* (Table 1 and Table S2). The pathway enrichment analysis showed that the sub-networks of 11 *PtHsfs* were enriched in 1–3 pathway(s), of which the “spliceosome pathway” was enriched in multiple sub-networks such as sub-networks of *PtHsf-A1b*, *PtHsf-A1c*, *PtHsf-A2*, *PtHsf-A5b* and *PtHsf-A7a* (Table 1 and Table S1), this implying PtHsfs might be involved in the process of alternative splicing. Altogether, the co-expressed network analysis of *PtHsfs* reveals that the members in the *PtHsf* gene family underwent functional divergence.

### 3.5. Alternative Splicing of *PtHsf*

Alternative splicing is an important post-transcriptional process and it will increase the functional diversity of proteins [40,41]. To further explore the functional divergence of *PtHsfs*, we then checked the alternative splicing events in the *PtHsf* gene family and their differences between paralogous pairs (Table 2). Based on the latest genome assembly and RNA-Seq short reads supports, six and four mRNA splice isoform transcripts were generated from *PtHsfA7a* and *PtHsfA7b*; respectively, three splice variants can be produced from *PtHsfA2*, *PtHsf-B2a* or *Pt-HsfB2b*; two alternative splicing transcripts can be expressed from *PtHsf-A1a*, *PtHsf-A1c*, *PtHsf-A4b*, *PtHsf-A4c*, *PtHsf-A6a*, *PtHsf-A8a*, *PtHsf-A8b*, *PtHsf-B2c* and *PtHsf-B4c*, respectively; and the remained 16 *PtHsfs* only produced one transcript (Table 2). Among the ten *PtHsf* paralogous pairs, five pairs can transcript at least two transcripts from each gene, of which *PtHsf-A1a/A1c*, *PtHsf-A4a/A4c*, *PtHsf-A8a/A8b* and *PtHsf-B2a/B2b* generated the same number of transcripts via distinct alternative splicing events (Table 2). *PtHsf-A6a/A6b*, *PtHsf-A7a/A7b* and *PtHsf-B4a/B4c* can produce a different number of splicing variants from each gene, respectively (Table 2). Whereas non-alternative splicing events were detected from the pairs of *PtHsf-A5a/A5b*, *PtHsf-B3a/B3b* and *PtHsf-B4b/B4d* based on the current database (Table 2). Noticeable, alternative splicing resultants were mainly the focus on a 3′-UTR modification which changes the mRNA stability and N-terminal deletion including DBD truncation and C-terminal deletion (Table 2). This result indicates that the asymmetric evolution of alternative splicing increased the protein complexity of the *PtHsf* gene family. 

### 3.6. The SNP Differences between *PtHsf* Paralogous Pairs

**Figure 5:**
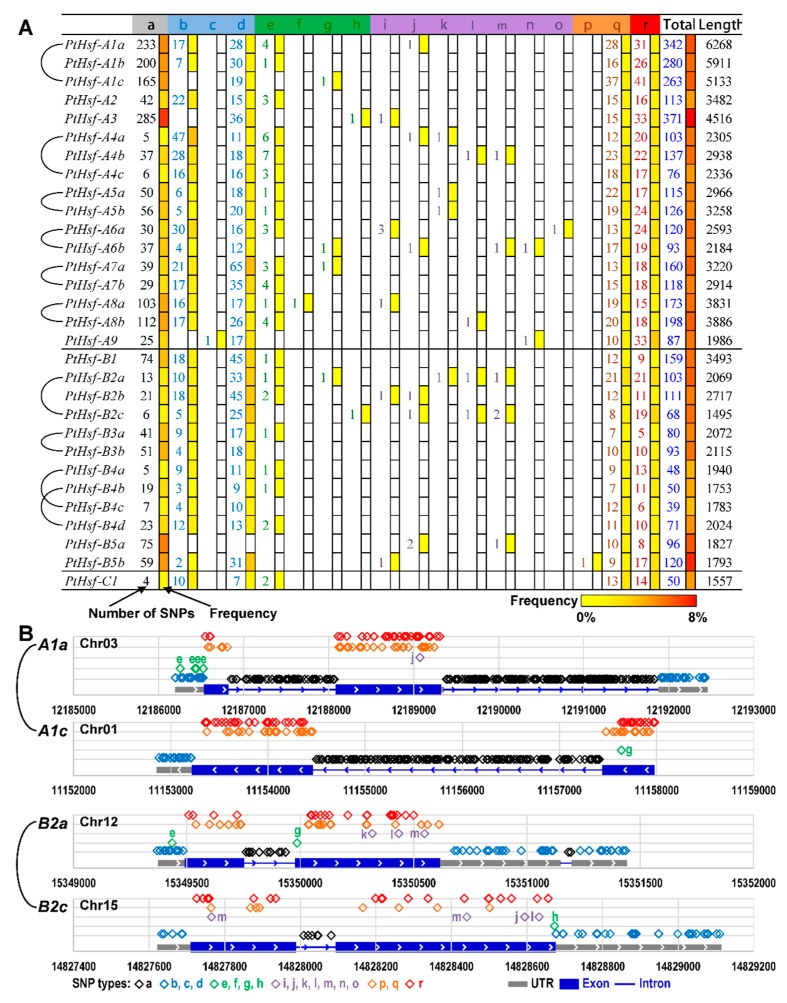
The identified single nucleotide polymorphisms (SNPs) in *PtHsf* genes from 549 *P. trichocarpa* individuals based on whole genome re-sequencing. (**A**) The SNPs were classified based on their locations: (a) intron, (b) UTR 5 prime, (c) UTR 3 deleted, (d) UTR 3 prime, (e) start gained, (f) start lost, (g) stop gained, (h) stop lost, (i) frame shift, (j) codon insertion, (k) codon deletion, (l) codon change plus codon deletion, (m) codon change plus codon insertion, (n) splice site donor, (o) splice site acceptor, (p) synonymous stop, (q) synonymous coding, (r) non-synonymous coding. (**B**) Examples of the SNP frequency and location of paralogous pairs *PtHsf-A1a/A1c* and *PtHsf-B2a/B2c*. Details of SNPs were listed in Table S3.

Single nucleotide polymorphism (SNP) is a way to produce gene variation that is also responsible for gene function, natural selection and genome evolution [42,43]. To investigate the natural variation in the *PtHsf* gene family, we analyzed the SNP dataset from a natural population that includes 549 *P*. *trichocarpa* individuals [22] (Figure 5, Table 3 and Table S3). The SNP density was significantly different in *PtHsf* genes, for example, a total of 342 SNPs were detected in *PtHsfA1a*, but only 50 SNPs were detected in its paralogs *PtHsf1c* (Figure 5A and Table S3). As shown in Figure 5A, SNPs can be divided into different types based on the location and consequence. SNPs in the *PtHsf* gene family were distributed in the intron, UTR region, and coding sequence that produce synonymous coding or non-synonymous coding (Figure 5, Table S3). In the SNPs types that produce protein variation, the frequency of non-synonymous coding and the start gained type of SNPs is much higher than other types such as the start lost, stop gained, stop lost and so on (Figure 5A, Table S3). Except for the protein coding region variation, the SNP frequency was higher in the regulatory regions such as 5′-UTR and 3′-UTR (Figure 5A), which adjusts the gene expression level under different environmental stresses. The SNP frequency of SNP types affecting the coding sequences of PtHsf proteins (such as the start gained and non-synonymous coding) varied greatly (Figure 5A, Table 3), even in *PtHsf-A4a/c* and *PtHsf-A4b/d*—the paralogous pairs with conserved expression and co-expressed genes (Figure 5A, Table 3). The SNP variation in *PtHsf* genes will increase the functional diversity and adaption to the environment at the population level. 

### 3.7. Divergence of Protein 3D Structure

**Figure 6:**
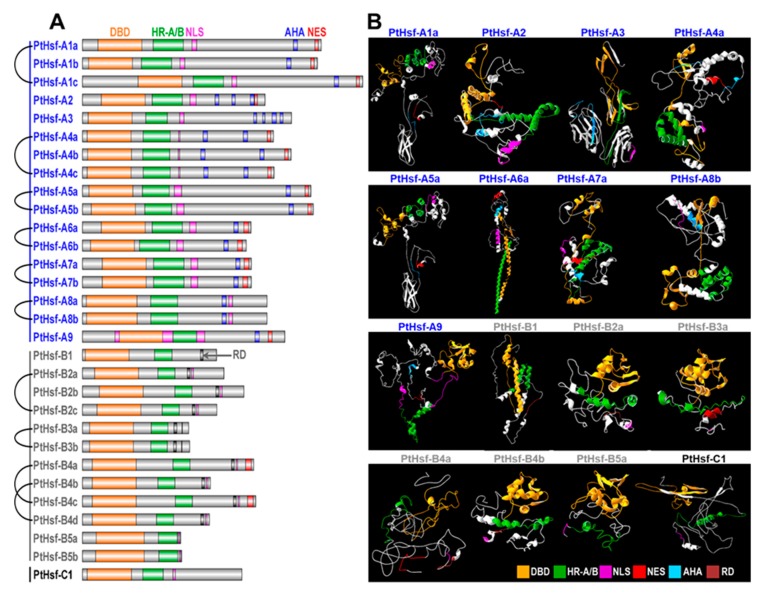
The conserved protein motifs and 3D structures of PtHsf proteins. (**A**) The diagram represents the motif domain distribution in PtHsfs. (**B**) Structural analysis of PtHsf proteins. Protein 3D structure of PtHsfs from each subfamily and each subclade were predicted.

PtHsfs were divided into three subclades based on the conserved domains. All the PtHsf proteins contained the DBD located in the N terminal and followed HR-A/B (Figure 6A). In the C terminal, the PtHsf-A subclade had the transcriptional activation motif (AHA motifs) which was lost in subclass B and C (Figure 6A), and subclass B PtHsf harbored the regulatory domain (RD). NLSs were located in subclass B and C PtHsf proteins and nuclear export signal (NES) were only found in the PtHsf-A subclass except for PtHsf-A8a/8b (Figure 6A). The composition and distribution of the conserved domains were similar to those previously reported in animals and *Arabidopsis* [25]. As a transcription factor, Hsf proteins played their roles via forming homotrimerization or heterotrimerization to interact with the DNA binding site and initiate the transaction of downstream target genes. So, it was very important for Hsf to maintain specific spatial configuration [44]. To predict the 3D structures, we selected 16 PtHsf proteins that covered each subfamily and each subclade for structural prediction (Figure 6B). The helix-turn-helix structure of DBD can be found in the N terminal except for PtHsf-A3, PtHsf-B4a and PtHsf-C1 (Figure 6B). The HR-A/B adjacent to DBD consisted of the α-helix, except for PtHsf-A3 and PtHsf-B4a (Figure 6B). This result means that the secondary structure of the N terminal of PtHsfs was conserved. In contrast, the C terminal showed secondary structure diversity (Figure 6B). For example, the NLS that was on the flanked of HR-A/B in subclade A formed an α-helix except for PtHsf-A3 and PtHsfA8b (Figure 6B); it formed a random coil in subclade B and C (Figure 6B). It is interesting that only PtHsf-A1a and PtHsf-A5a shared a similar geometry of packing in the PtHsf-A subclade (Figure 6A), which was similar to human Hsf1 and Hsf2 proteins [44]. In the PtHsf-B subclade, PtHsf-B2a, PtHsf-B3a, PtHsf-B4b and PtHsf-B5a had similar geometries of packing, but the length and position of the α-coil in the HR-A/B domain were different in the four proteins (Figure 6B). We then compared the 3D structure of paralogous pairs (Figure S1) and found that there were divergences of the tertiary structure between the paralogous pairs of PtHsf-A5a/A5b, PtHsf-Aa6a/A6b, PtHsf-A7a/A7b, PtHsf-A8a/A8b, PtHsf-B2a/B2c, and PtHsf-B4a/B4c (Figure S1). Altogether, PtHsf members retained conserved functional motifs but had a tertiary structure divergence.

## 4. Discussion

Hsf transcription factors are direct upstream regulators of *Hsp* genes by interacting with their *cis*-acting elements HSE, it plays the diversity function in plant development and responds to various environmental stresses [1]. The plant evolved *Hsf* genes from unicellular algae and underwent gene expansion via whole genome duplication (Figure 1) [14]. However, how the *PtHsf* gene family evolved after whole-genome duplication events is unclear. In this study, we found that the expression pattern of *PtHsf* paralogous pairs was positively correlated with promoter region conservation. Furthermore, we found that the alternative splicing, SNP distribution and frequency, and protein 3D structure were divergent in *PtHsfs* paralogous pairs, implying that the *Hsf* gene family acquired multiple ways to increase their protein diversity to adapt to the environment. This study comprehensively analyzed the potential functional divergence of *PtHsf* genes, uncovering the possible evolutionary history of the *PtHsf* gene family in *Populus*.

Gene functional diversification accelerated by gene expansion promotes the environmental adaption ability of an organism [45]. In *Populus*, the *PtHsf* gene family underwent gene expansion during evolution (Figure 1) [12]. As key regulators of the heat shock response, seven *PtHsfs* were validated to respond to heat shock and activate *Hsps* expression in poplar [12]. Moreover, the *PtHsf* gene family had a diversity expression pattern in different organs [12], different biological processes and nitrogen response (Figure 2A), which suggests that the *PtHsf* gene family might acquire new functions in the development and nitrogen response from gene expansion process. The expression pattern diversity of the *Hsf* gene family was widely reported in *Arabidopsis*, rice, and so on [46]. In our co-expression network, only *PtHsf-B4a/4c*, *PtHsf-B4b/4d*, and *PtHsf-A3a/3b* were located in the same sub-network, respectively, others were in relatively independent sub-networks (Figure 4). The GO enrichment analysis showed that the *PtHsf* gene expression diversity was consistent with their potential function (Table S1). For example, the *PtHsf-A1b* sub-network was enriched in catkins development related processes (Table S1) [38]. The sub-networks of *PtHsf-B4a/B4c* and *PtHsf-B4b/B4d* dynamically regulated in leaf development were enriched in terms of cell construction and the photosynthetic electron transport chain, respectively (Table S1). Sub-networks of *PtHsfs* that responded to different types of nitrogen treatment were also enriched in nitrogen compound metabolic and biosynthetic processes (Table S1). Because the mRNA abundance of functionally related genes are coordinated regulated [37], *PtHsf* co-expression network analysis and GO enrichment further indicated that neofunctionalization occurred in the *PtHsf* gene family. A similar function of GO terms was the enrichment in the sub-networks of *PtHsf-A1b*, *PtHsf-A1c*, *PtHsf-A2*, *PtHsf-A5b*, *PtHsf-B4a* and *PtHsf-B4c* (Figure 4B), which might be caused by functional redundancy. However, they showed differences in the enriched domain and co-expressed TFs (Table S1). *PtHsf-B4b* and *PtHsf-B4d* were the most conserved paralogous genes in the *PtHsf* gene family because of the similar expression pattern, GO enrichment and domain enrichment (Figure 4B, Table S1). In the co-expressed TFs, two members from the HD-ZIP family were co-expressed with both *PtHsf-B4b* and *PtHsf-B4d* (Table 1 and Table S2), one of which is a homolog of *PHB* which is involved in the polarity establishment in leaf primordium [47]. Noticeably, *PtHsf-B4d* was specifically co-expressed with two *TCP* TFs, which were reported to be affecting the leaf shape in *Arabidopsis* [48,49]. This specific co-expression might imply the specific role of *PtHsf-B4d* in a specific stage/process of leaf development. Altogether, the members of the *PtHsf* gene family showed functional diversity.

The gene promoter determines its expression pattern, we found that the expression similarity of *PtHsf* paralogous pairs was positively correlated with the length of the conserved promoter region (Figure 3). In addition, the promoter choice can influence the splice site selection [50,51]. In our study, we found that the *PtHsf* genes had various alternative splicing isoforms (Table 1), even in the paralogous pair *PtHsf-B4a/4c* with a similar expression pattern (Figure 2 and Table 2). Noticeably, *PtHsf-B4b/4d* and *PtHsf-A3a/3b* were paralogous pairs with more than 400 bp conserved blocks close to the translation initiation site that only produced one transcript from each gene; other paralogous pairs with a relatively low conservation in the promoter region produced a diverse splicing isoform except for *PtHsf-A5a/5c* (Figure 3, Table 1). The different alternative splicing might be caused by the difference in 5′-UTR. It has been reported that the sequence and secondary structure of the 5′-flanking region plays a key role in determining the alternative splicing site [50,51]. In human hepatoma Hep3B cells, a five-point mutation out from a 220 bp promoter can change the splicing pattern [50]. So, the promoter modification might be one of the reasons for the alternative splicing diversity in the *PtHsf* gene family. Another reason is that the unbalanced alternative splicing isoform might be related to the co-expressed genes in the “spliceosome pathway” (Table S1). Sub-networks of *PtHsf-A1c*, *PtHsf-A2*, and *PtHsf-A7a* was enriched in the “spliceosome pathway”; these genes had at least two transcripts (Table 2). Furthermore, the unevenly distributed SNPs in *PtHsf* genes may also mediate the difference of alternative splicing (Figure 5). In humans, the *MYLK* gene with two SNPs in intron eleven generated a splicing variant because the presence of two SNPs at the acceptor site affected the recognition of the spliceosome [52]. In the *Arabidopsis* Wassilewskia accession, *RPT5b* harbors an SNP in intron seven, producing a mis-splicing transcript [53]. Based on the *P. trichocarpa* population, we identified one SNP at the splice donor site of *PtHsf-A6a* and *PtHsf-A9* and one SNP at the splice acceptor site of *PtHsf-A6b* (Figure 5), which may influence their alternative splicing process in specific genotypes (Figure 5). Altogether, the promoter divergence, co-expressed genes and SNPs might contribute to the alternative splicing of *PtHsf* genes in different levels, these regulatory mechanisms increased the functional diversity of *PtHsfs*. 

The protein 3D structure is pivotal for the functional maintenance of proteins. We found that the N-terminal of PtHsfs were conserved in the sequence identity and secondary structure (Figure 6). The winged helix-turn-helix were formed in DBD which will interact with a major groove of the DNA (Figure 6B) [44,54], and the α-helix also shaped in the HR-A/B were responses for trimerization when Hsf was activated (Figure 6B) [54]. The secondary structures of DBD and HR-A/B of PtHsfs were similar to animal Hsfs [44,54]. We also found that the structure of the C-terminal of PtHsf was more diverse than the N-terminal (Figure 6B). This is consistent with the sequence identified among the 16 PtHsfs was below 35% (Table S4), which is a marginal value for the protein structure similarity [55]. The paralogous pairs were generated from genome duplication events, the protein 3D structure should be conserved; but we noticed that the tertiary structures were divergent in PtHsf paralogous pairs (Figure S1). It might be affected by specific amino-acid residue mutations [56,57]. There were 11 and 6 amino-acid residue mutants in the Pro or Gly which were helix-breaking residues [57] when comparing the protein sequence of PtHsf-B2a/B2c and PtHsf-A8a/A8b, respectively (Figure S2). This is consistent with the protein 3D structures, PtHsf-B2c and PtHsf-A8a, which have a long α-helix while PtHsf-B2a and PtHsf-A8b consisted of a short α-helix and random coil (Figure S1). Above all, both sequence identities and amino-acid residue mutations may affect the protein 3D structure divergence in the PtHsf gene family. 

## 5. Conclusions

In this study, we found that the *Hsf* gene family was a conserved family across the world and expanded in plants via genome duplication. Furthermore, the functional diversity of *PtHsfs* was evaluated by expression profiles and co-expressed gene enrichment. In addition, the modification of the promoter and coding sequence region led to the divergence of alternative splicing events and the 3D structure. This study comprehensively analyzed the potential functional diversity of *PtHsf* genes and uncovered the evolutionary history of the *PtHsf* gene family. Further investigations of the function of the *PtHsf* gene family in poplar will uncover the molecular mechanism of *PtHsfs* in poplar development and adaption to dynamic environments.

## Figures and Tables

**Table 1:** The co-expressed genes and enrichment analysis.

Gene Name	Co-Expressed Gene #	Co-Expressed TF #	Enriched GO Term	Enriched Protein Domain	Enriched Pathway
*PtHsf-A1a*	268	8	9	9	1
*PtHsf-A1b*	281	2	17	20	1
*PtHsf-A1c*	458	21	57	2	1
*PtHsf-A2*	439	6	16	41	2
*PtHsf-A3*	84	3	n.a.	2	n.a.
*PtHsf-A4a*	275	24	n.a.	5	n.a.
*PtHsf-A4b*	17	4	n.a.	n.a.	n.a.
*PtHsf-A4c*	0	0	n.a.	n.a.	n.a.
*PtHsf-A5a*	137	4	n.a.	n.a.	n.a.
*PtHsf-A5b*	626	21	56	28	1
*PtHsf-A6a*	6	0	n.a.	n.a.	n.a.
*PtHsf-A6b*	0	0	n.a.	n.a.	n.a.
*PtHsf-A7a*	91	4	n.a.	13	1
*PtHsf-A7b*	54	3	n.a.	n.a.	n.a.
*PtHsf-A8a*	8	1	n.a.	n.a.	n.a.
*PtHsf-A8b*	17	2	n.a.	n.a.	n.a.
*PtHsf-A9*	15	2	n.a.	n.a.	n.a.
*PtHsf-B1*	269	11	19	1	1
*PtHsf-B2a*	18	0	n.a.	6	1
*PtHsf-B2b*	0	0	n.a.	n.a.	n.a.
*PtHsf-B2c*	39	3	n.a.	n.a.	n.a.
*PtHsf-B3a*	27	1	n.a.	n.a.	n.a.
*PtHsf-B3b*	103	5	n.a.	2	n.a.
*PtHsf-B4a*	315	10	53	n.a.	1
*PtHsf-B4b*	176	6	7	11	3
*PtHsf-B4c*	391	29	59	5	n.a.
*PtHsf-B4d*	200	8	6	11	3
*PtHsf-B5a*	0	0	n.a.	n.a.	n.a.
*PtHsf-B5b*	154	12	n.a.	2	n.a.
*PtHsf-C1*	2	1	n.a.	n.a.	n.a.

n.a.: no significant term available.

**Table 2:** The gene list and corresponding transcripts of *PtHsfs*.

Class	Gene Name	Gene ID	Transcript ID	AS type ^a^	Resultant
Class A	*PtHsf-A1a*	Potri.003G095000	Potri.003G095000.1	(primary transcript)	
			Potri.003G095000.2	A5SS	3′ UTR change
	*PtHsf-A1b*	Potri.013G079800	Potri.013G079800.1	(primary transcript)	
	*PtHsf-A1c*	Potri.001G138900	Potri.001G138900.3	(primary transcript)	
			Potri.001G138900.2	A3SS	loss partial of DBD
	*PtHsf-A2*	Potri.006G226800	Potri.006G226800.4	(primary transcript)	
			Potri.006G226800.2	A5SS, SE	loss partial of C-terminal and 3′ UTR change
			Potri.006G226800.3	A5SS	loss partial of C-terminal and 3′ UTR change
	*PtHsf-A3*	Potri.006G115700	Potri.006G115700.2	(primary transcript)	
	*PtHsf-A4a*	Potri.011G071700	Potri.011G071700.1	(primary transcript)	
	*PtHsf-A4b*	Potri.014G141400	Potri.014G141400.1	(primary transcript)	
			Potri.014G141400.2	RI	loss DBD
	*PtHsf-A4c*	Potri.004G062300	Potri.004G062300.1	(primary transcript)	
			Potri.004G062300.2	A3SS	loss partial DBD
	*PtHsf-A5a*	Potri.017G059600	Potri.017G059600.1	(primary transcript)	
	*PtHsf-A5b*	Potri.001G320900	Potri.001G320900.1	(primary transcript)	
	*PtHsf-A6a*	Potri.010G082000	Potri.010G082000.2	(primary transcript)	
			Potri.010G082000.1	SE	3′ UTR change
	*PtHsf-A6b*	Potri.008G157600	Potri.008G157600.1	(primary transcript)	
	*PtHsf-A7a*	Potri.005G214800	Potri.005G214800.2	(primary transcript)	
			Potri.005G214800.4	SE, SE	3′ UTR change
			Potri.005G214800.1	SE	3′ UTR change
			Potri.005G214800.3	SE, SE, SE	3′ UTR change
			Potri.005G214800.5	SE, SE	3′ UTR change
			Potri.005G214800.6	RI	N-terminal
	*PtHsf-A7b*	Potri.002G048200	Potri.002G048200.1	(primary transcript)	
			Potri.002G048200.2	RI	3′ UTR change
			Potri.002G048200.3	SE	3′ UTR change
			Potri.002G048200.4	RI, SE	3′ UTR change
	*PtHsf-A8a*	Potri.008G136800	Potri.008G136800.2	(primary transcript)	
			Potri.008G136800.6	A5SS	3′ UTR change
	*PtHsf-A8b*	Potri.010G104300	Potri.010G104300.2	(primary transcript)	
			Potri.010G104300.1	SE	3′ UTR change
	*PtHsf-A9*	Potri.006G148200	Potri.006G148200.2	(primary transcript)	
Class B	*PtHsf-B1*	Potri.007G043800	Potri.007G043800.1	(primary transcript)	
	*PtHsf-B2a*	Potri.012G138900	Potri.012G138900.1	(primary transcript)	
			Potri.012G138900.2	SE, RI	3′ UTR change
			Potri.012G138900.3	RI	3′ UTR change
	*PtHsf-B2b*	Potri.001G108100	Potri.001G108100.1	(primary transcript)	
			Potri.001G108100.3	A3SS	5′ UTR change
			Potri.001G108100.2	A5SS	5′ UTR change
	*PtHsf-B2c*	Potri.015G141100	Potri.015G141100.1	(primary transcript)	
			Potri.015G141100.2	SE	loss partial of internal sequence
	*PtHsf-B3a*	Potri.006G049200	Potri.006G049200.1	(primary transcript)	
	*PtHsf-B3b*	Potri.016G056500	Potri.016G056500.1	(primary transcript)	
	*PtHsf-B4a*	Potri.002G124800	Potri.002G124800.1	(primary transcript)	
	*PtHsf-B4b*	Potri.009G068000	Potri.009G068000.1	(primary transcript)	
	*PtHsf-B4c*	Potri.014G027100	Potri.014G027100.3	(primary transcript)	
			Potri.014G027100.1	RI	loss partial of C-terminal
	*PtHsf-B4d*	Potri.001G273700	Potri.001G273700.1	(primary transcript)	
	*PtHsf-B5a*	Potri.004G042600	Potri.004G042600.1	(primary transcript)	
	*PtHsf-B5b*	Potri.011G051600	Potri.011G051600.1	(primary transcript)	
Class C	*PtHsf-C1*	Potri.T137400	Potri.T137400.1	(primary transcript)	

^a^: Alternative splicing types include skipped exon (SE), alternative 5′ splice sites (A5SS), alternative 3′ splice sites (A3SS), mutually exclusive exons (MXE), and retained intron (RI).

**Table 3:** The non-synonymous coding single nucleotide polymorphisms (SNPs) located in the domain of *PtHsfs*.

Gene Name	DBD	HR-A/B	AHA	Others (NLS, NES, or RD)
***PtHsf-A1a***	Chr03:12188111 G->T (W105C)	Chr03:12188394 A->G (M200V)		
	Chr03:12188143 A->T (Q116L)			
***PtHsf-A1b***	Chr13:6946101 T->C (K107E)	Chr13:6946028 G->A (S131F)		Chr13:6945777 T->G (K215Q) <NLS>
		Chr13:6945966 C->A (V152F)		
		Chr13:6945909 G->C (R171G)		
***PtHsf-A1c***	Chr01:11154369 C->T (R211Q)	Chr01:11154258 T->C (E248G)	Chr01:11153373 G->A (A543V)	
		Chr01:11154213 C->T (R263K)		
		Chr01:11154182 G->T (S273R)		
***PtHsf-A2***	Chr06:23831182 C->A (S94I)	Chr06:23830387 C->G (M171I)		
	Chr06:23830564 C->A (W112C)	Chr06:23830281 T->C (T207A)		
***PtHsf-A3***	Chr06:9090513 G->T (P15H)	Chr06:9088646 C->T (V367I)		
	Chr06:9090444 A->G (I38T)	Chr06:9088592 G->A (P385S)		
	Chr06:9089476 G->A (S90F)			
	Chr06:9089434 C->A (R104M)			
***PtHsf-A4a***	Chr11:6830049 T->A (N37Y)	Chr11:6829640 C->T (D132N)		Chr11:6828857 G->A (L393F) <NES>
	Chr11:6830025 G->T (P45T)	Chr11:6829620 T->A (K138N)		
		Chr11:6829607 C->A (A143S)		
		Chr11:6829480 C->G (R185P)		
		Chr11:6829475 A->T (L187M)		
***PtHsf-A4b***	Chr14:10767449 A->C (S6R)	Chr14:10766450 T->C (K131R)		Chr14:10765533 T->C (I437V) <NES>
	Chr14:10766596 C->A (Q82H)	Chr14:10766449 C->A (K131N)		
	Chr14:10766553 T->G (I97L)	Chr14:10766399 T->C (Q148R)		
		Chr14:10766333 C->G (S170T)		
		Chr14:10766295 C->A (G183C)		
		Chr14:10766294 C->G (G183A)		
***PtHsf-A4c***	Chr04:5146215 G->A (P25L)	Chr04:5145571 T->A (M187L)	Chr04:5145100 C->G (V344L)	
	Chr04:5146204 G->A (P29S)			
	Chr04:5146203 G->T (P29Q)			
	Chr04:5146185 T->G (Q35P)			
	Chr04:5146182 C->G (S36T)			
***PtHsf-A5a***	Chr17:5478669 C->T (S43N)	Chr17:5477318 G->C (H138D)		Chr17:5476292 T->C (M480V) <NES>
	Chr17:5477446 T->C (K95R)	Chr17:5477236 T->A (Q165L)		
		Chr17:5477207 C->T (E175K)		
***PtHsf-A5b***	Chr01:32571330 G->T (F23L)	Chr01:32569699 T->C (K144R)		
	Chr01:32571193 T->C (N69S)	Chr01:32569688 T->C (K148E)		
	Chr01:32569818 G->T (H104Q)	Chr01:32569582 A->C (L183R)		
***PtHsf-A6a***	Chr10:10826222 C->A (S64I)	Chr10:10825317 G->C (L156V)		Chr10:10825077 T->A (I236F) <NLS>
	Chr10:10826111 G->T (T101K)	Chr10:10825283 T->C (K167R)		Chr10:10824747 A->C (L346V) <NES>
	Chr10:10825416 T->C (K123E)	Chr10:10825281 G->T (Q168K)		Chr10:10824724 C->G (L353F) <NES>
		Chr10:10825251 T->C (R178G)		
		Chr10:10825200 C->T (V195I)		
		Chr10:10825148 G->A (A212V)		
***PtHsf-A6b***	Chr08:10686052 C->T (P18S)	Chr08:10686988 T->A (L129Q)		Chr08:10687618 A->C (Y339S) <NES>
		Chr08:10687036 T->C (V145A)		
		Chr08:10687083 A->C (I161L)		
		Chr08:10687099 G->A (R166Q)		
		Chr08:10687140 A->G (S180G)		
***PtHsf-A7a***	Chr05:22774061 G->T (A74S)	Chr05:22774833 G->T (R156L)		Chr05:22775403 C->T (T346I) <NES>
	Chr05:22774724 G->A (G120R)	Chr05:22774866 G->A (R167K)		
		Chr05:22774951 C->G (D195E)		
		Chr05:22774954 A->C (Q196H)		
***PtHsf-A7b***	Chr02:3141391 T->C (D56G)	Chr02:3140451 G->C (H171Q)		Chr02:3140246 T->A (T240S) <NLS>
	Chr02:3141341 C->T (V73I)	Chr02:3140405 C->T (A187T)		
	Chr02:3141326 A->G (Y78H)	Chr02:3140318 T->A (M216L)		
	Chr02:3141309 A->T (N83K)			
	Chr02:3140561 T->C (R135G)			
***PtHsf-A8a***				Chr08:9112715 G->A (E251K) <NLS>
***PtHsf-A8b***	Chr10:12489151 T->G (M19L)			
	Chr10:12489048 T->C (K53R)			
	Chr10:12487083 T->A (D78V)			
	Chr10:12487082 A->T (D78E)			
	Chr10:12487060 C->T (G86R)			
	Chr10:12486987 C->T (R110Q)			
***PtHsf-A9***	Chr06:12750969 T->C (K103E)	Chr06:12750442 C->G (V206L)		Chr06:12750381 C->T (S226N) <NLS>
	Chr06:12750927 A->G (S117P)	Chr06:12750409 T->G (K217Q)		
	Chr06:12750921 T->C (N119D)			
	Chr06:12750920 T->A (N119I)			
	Chr06:12750618 G->A (P147L)			
***PtHsf-B1***		Chr07:3779417 T->A (E174V)		
		Chr07:3779389 C->A (L183F)		
***PtHsf-B2a***	Chr12:15349619 G->A (D44N)	Chr12:15350210 G->T (E166D)		Chr12:15350412 G->A (G234R) <NLS>
	Chr12:15349721 T->C (F78L)	Chr12:15350293 C->G (S194W)		Chr12:15350397 G->T (V229F) <RD>
	Chr12:15350046 T->A (L112M)			
	Chr12:15350051 A->T (L113F)			
	Chr12:15350067 A->G (R119G)			
***PtHsf-B2b***	Chr01:8582316 C->G (D55E)	Chr01:8582880 C->T (S209L)		Chr01:8583138 T->C (V295A) <NLS>
	Chr01:8582335 G->A (D62N)			
	Chr01:8582651 A->C (T133P)			
***PtHsf-B2c***	Chr15:14827877 A->T (E56D)	Chr15:14828338 A->G (E175G)		Chr15:14828531 G->T (E239D) <RD>
	Chr15:14827921 G->T (R71I)			
	Chr15:14827935 A->C (K76Q)			
***PtHsf-B3a***		Chr06:3535947 A->G (K152R)		
		Chr06:3535970 G->T (V160F)		
		Chr06:3535988 A->T (T166S)		
***PtHsf-B3b***	Chr16:3748780 T->A (I36L)	Chr16:3747556 G->T (T162N)		
	Chr16:3748759 T->C (T43A)	Chr16:3747537 G->C (N168K)		
	Chr16:3748734 G->T (A51E)	Chr16:3747526 T->C (K172R)		
	Chr16:3747757 C->A (R95L)			
***PtHsf-B4a***	Chr02:9388712 C->G (T49S)	Chr02:9389317 T->C (S209P)		Chr02:9389755 G->C (G355R) <NES>
	Chr02:9388795 G->T (V77F)	Chr02:9389350 G->A (D220N)		Chr02:9389656 T->C (S322P) <RD>
***PtHsf-B4b***	Chr09:6774240 C->A (L80I)	Chr09:6774986 A->C (K167Q)		Chr09:6775257 G->A (G257E) <RD>
				Chr09:6775259 G->T (V258F) <RD>
***PtHsf-B4c***		Chr14:2300725 A->C (M211L)		Chr14:2301145 A->T (M351L) <NES>
				Chr14:2301156 C->A (D354E) <NES>
***PtHsf-B4d***	Chr01:28036766 T->G (N81K)	Chr01:28037553 A->C (N169H)		
	Chr01:28037311 T->C (V88A)			
	Chr01:28037358 G->A (A104T)			
***PtHsf-B5a***	Chr04:3233937 G->T (P53H)	Chr04:3232326 G->C (T195S)		
	Chr04:3232591 A->T (S107T)			
	Chr04:3232540 A->T (L124M)			
***PtHsf-B5b***	Chr11:4423922 C->A (A45S)	Chr11:4422794 G->A (T162M)		
	Chr11:4423915 T->C (D47G)	Chr11:4422692 G->T (T196N)		
	Chr11:4423904 C->A (D51Y)	Chr11:4422679 T->C (I200M)		
	Chr11:4423865 C->T (E64K)			
	Chr11:4423834 G->T (A74D)			
	Chr11:4423825 G->A (S77L)			
	Chr11:4422971 T->C (K103R)			
	Chr11:4422954 G->T (Q109K)			
	Chr11:4422936 C->T (E115K)			
	Chr11:4422931 C->A (K116N)			
***PtHsf-C1***		scaffold_294:25641 C->G (G149A)		

## Supplementary Materials

The following are available online at https://www.mdpi.com/2073-4409/8/5/438/s1, Figure S1. 3D structures comparison of paralogous pairs from PtHsf family. Figure S2. Two paralogous pairs protein sequence alignment analysis. Table S1. Enrichment analysis of PtHsfs co-expressed genes. Table S2. List of transcription factor genes co-expressed with PtHsfs. Table S3. SNP information of PtHsf gene family. Table S4. protein sequence similarity in Figure 6B.

## References

[1] Jacob P., Hirt H., Bendahmane A. (2017). The heat-shock protein/chaperone network and multiple stress resistance. Plant Biotechnol. J..

[2] Akerfelt M., Morimoto R.I., Sistonen L. (2010). Heat shock factors: Integrators of cell stress, development and lifespan. Nat. Rev. Mol. Cell Biol..

[3] Lindquist S. (1986). The heat-shock response. Ann. Rev. Biochem..

[4] Zhang J., Li J., Liu B., Zhang L., Chen J., Lu M. (2013). Genome-wide analysis of the populus hsp90 gene family reveals differential expression patterns, localization, and heat stress responses. BMC Genom..

[5] Anckar J., Sistonen L. (2011). Regulation of hsf1 function in the heat stress response: Implications in aging and disease. Ann. Rev. Biochem..

[6] Jedlicka P., Mortin M.A., Wu C. (1997). Multiple functions of drosophila heat shock transcription factor In Vivo. EMBO J..

[7] Liu H.C., Charng Y.Y. (2013). Common and distinct functions of arabidopsis class a1 and a2 heat shock factors in diverse abiotic stress responses and development. Plant Physiol..

[8] Wu C. (1984). Activating protein factor binds in vitro to upstream control sequences in heat shock gene chromatin. Nature.

[9] Gomez-Pastor R., Burchfiel E.T., Thiele D.J. (2018). Regulation of heat shock transcription factors and their roles in physiology and disease. Nat. Rev. Mol. Cell Biol..

[10] Nover L., Bharti K., Doring P., Mishra S.K., Ganguli A., Scharf K.D. (2001). Arabidopsis and the heat stress transcription factor world: How many heat stress transcription factors do we need?. Cell Stress Chaperones.

[11] Zhang J., Li Y., Jia H.X., Li J.B., Huang J., Lu M.Z., Hu J.J. (2015). The heat shock factor gene family in salix suchowensis: A genome-wide survey and expression profiling during development and abiotic stresses. Front. Plant Sci..

[12] Zhang J., Liu B., Li J., Zhang L., Wang Y., Zheng H., Lu M., Chen J. (2015). Hsf and hsp gene families in populus: Genome-wide identification, organization and correlated expression during development and in stress responses. BMC Genom..

[13] Zhang J., Jia H., Li J., Li Y., Lu M., Hu J. (2016). Molecular evolution and expression divergence of the populus euphratica hsf genes provide insight into the stress acclimation of desert poplar. Sci. Rep..

[14] Wang X., Shi X., Chen S., Ma C., Xu S. (2018). Evolutionary origin, gradual accumulation and functional divergence of heat shock factor gene family with plant evolution. Front. Plant Sci..

[15] Taylor G. (2002). Populus: Arabidopsis for forestry. Do we need a model tree?. Ann. Bot..

[16] Gordon J.C. (2001). Poplars: Trees of the people, trees of the future. For. Chron..

[17] Demura T., Ye Z.H. (2010). Regulation of plant biomass production. Curr. Opin. Plant Biol..

[18] Sannigrahi P., Ragauskas A.J., Tuskan G.A. (2010). Poplar as a feedstock for biofuels: A review of compositional characteristics. Biofuels Bioprod. Biorefining.

[19] Wilkinson A. (1999). Poplars and willows for soil erosion control in new zealand. Biomass Bioenergy.

[20] Tuskan G.A., Difazio S., Jansson S., Bohlmann J., Grigoriev I., Hellsten U., Putnam N., Ralph S., Rombauts S., Salamov A. (2006). The genome of black cottonwood, populus trichocarpa (torr. & gray). Science.

[21] Jansson S., Douglas C.J. (2007). Populus: A model system for plant biology. Ann. Rev. Plant Biol..

[22] Evans L.M., Slavov G.T., Rodgers-Melnick E., Martin J., Ranjan P., Muchero W., Brunner A.M., Schackwitz W., Gunter L., Chen J.G. (2014). Population genomics of populus trichocarpa identifies signatures of selection and adaptive trait associations. Nat. Genet..

[23] Oubida R.W., Gantulga D., Zhang M., Zhou L., Bawa R., Holliday J.A. (2015). Partitioning of multivariate phenotypes using regression trees reveals complex patterns of adaptation to climate across the range of black cottonwood (populus trichocarpa). Front. Plant Sci..

[24] McKown A.D., Guy R.D., Klapste J., Geraldes A., Friedmann M., Cronk Q.C., El-Kassaby Y.A., Mansfield S.D., Douglas C.J. (2014). Geographical and environmental gradients shape phenotypic trait variation and genetic structure in populus trichocarpa. New Phytol..

[25] Kotak S., Port M., Ganguli A., Bicker F., Von Koskull-Döring P. (2004). Characterization of c-terminal domains of arabidopsis heat stress transcription factors (hsfs) and identification of a new signature combination of plant class a hsfs with aha and nes motifs essential for activator function and intracellular localization. Plant J..

[26] Lozano R., Hamblin M.T., Prochnik S., Jannink J.L. (2015). Identification and distribution of the nbs-lrr gene family in the cassava genome. BMC Genom..

[27] Trifinopoulos J., Nguyen L.T., Minh B.Q., von Haeseler A. (2016). W-iq-tree: A fast online phylogenetic tool for maximum likelihood analysis. Nucleic Acids Res..

[28] Kalyaanamoorthy S., Minh B.Q., Wong T.K.F., von Haeseler A., Jermiin L.S. (2017). Modelfinder: Fast model selection for accurate phylogenetic estimates. Nat. Methods.

[29] Hoang D.T., Chernomor O., von Haeseler A., Minh B.Q., Vinh L.S. (2018). Ufboot2: Improving the ultrafast bootstrap approximation. Mol. Biol. Evol..

[30] Letunic I., Bork P. (2016). Interactive tree of life (itol) v3: An online tool for the display and annotation of phylogenetic and other trees. Nucleic Acids Res..

[31] Chow C.N., Lee T.Y., Hung Y.C., Li G.Z., Tseng K.C., Liu Y.H., Kuo P.L., Zheng H.Q., Chang W.C. (2018). Plantpan3. 0: A new and updated resource for reconstructing transcriptional regulatory networks from chip-seq experiments in plants. Nucleic Acids Res..

[32] Smoot M.E., Ono K., Ruscheinski J., Wang P.L., Ideker T. (2010). Cytoscape 2.8: New features for data integration and network visualization. Bioinformatics.

[33] Yang J., Yan R., Roy A., Xu D., Poisson J., Zhang Y. (2015). The i-tasser suite: Protein structure and function prediction. Nat. Methods.

[34] Project A.G. (2013). The amborella genome and the evolution of flowering plants. Science.

[35] Cooke J.E., Eriksson M.E., Junttila O. (2012). The dynamic nature of bud dormancy in trees: Environmental control and molecular mechanisms. Plant Cell Environ..

[36] Rinne P.L., Kaikuranta P.M., van der Schoot C. (2001). The shoot apical meristem restores its symplasmic organization during chilling-induced release from dormancy. Plant J..

[37] Ruprecht C., Vaid N., Proost S., Persson S., Mutwil M. (2017). Beyond genomics: Studying evolution with gene coexpression networks. Trends Plant Sci..

[38] Reddy T.V., Kaur J., Agashe B., Sundaresan V., Siddiqi I. (2003). The duet gene is necessary for chromosome organization and progression during male meiosis in arabidopsis and encodes a phd finger protein. Development.

[39] Volkenburgh E.V. (1999). Leaf expansion–an integrating plant behaviour. Plant Cell Environ..

[40] Iniguez L.P., Hernandez G. (2017). The evolutionary relationship between alternative splicing and gene duplication. Front. Genet..

[41] Su Z., Wang J., Yu J., Huang X., Gu X. (2006). Evolution of alternative splicing after gene duplication. Genome Res..

[42] Shastry B.S. (2009). Snps: Impact on gene function and phenotype. Methods Mol. Biol..

[43] Shastry B.S. (2002). Snp alleles in human disease and evolution. J. Hum. Genet..

[44] Jaeger A.M., Pemble C.W.T., Sistonen L., Thiele D.J. (2016). Structures of hsf2 reveal mechanisms for differential regulation of human heat-shock factors. Nat. Struct. Mol. Biol..

[45] Dunn M.J., Kinney G.M., Washington P.M., Berman J., Anderson M.Z. (2018). Functional diversification accompanies gene family expansion of med2 homologs in candida albicans. PLoS Genet..

[46] Guo M., Liu J.H., Ma X., Luo D.X., Gong Z.H., Lu M.H. (2016). The plant heat stress transcription factors (hsfs): Structure, regulation, and function in response to abiotic stresses. Front. Plant Sci..

[47] McConnell J.R., Emery J., Eshed Y., Bao N., Bowman J., Barton M.K. (2001). Role of phabulosa and phavoluta in determining radial patterning in shoots. Nature.

[48] Aguilar-Martinez J.A., Sinha N. (2013). Analysis of the role of arabidopsis class i tcp genes attcp7, attcp8, attcp22, and attcp23 in leaf development. Front. Plant Sci..

[49] Kieffer M., Master V., Waites R., Davies B. (2011). Tcp14 and tcp15 affect internode length and leaf shape in arabidopsis. Plant J..

[50] Cramer P., Pesce C.G., Baralle F.E., Kornblihtt A.R. (1997). Functional association between promoter structure and transcript alternative splicing. Proc. Natl. Acad. Sci. USA.

[51] Pecci A., Viegas L.R., Baranao J.L., Beato M. (2001). Promoter choice influences alternative splicing and determines the balance of isoforms expressed from the mouse bcl-x gene. J. Biol. Chem..

[52] Mascarenhas J.B., Tchourbanov A.Y., Fan H., Danilov S.M., Wang T., Garcia J.G. (2017). Mechanical stress and single nucleotide variants regulate alternative splicing of the mylk gene. Am. J. Resp. Cell Mol..

[53] Guyon-Debast A., Lécureuil A., Bonhomme S., Guerche P., Gallois J.L. (2010). A snp associated with alternative splicing of rpt5b causes unequal redundancy between rpt5a and rpt5b among arabidopsis thaliana natural variation. BMC Plant Biol..

[54] Takii R., Fujimoto M. (2016). Structure and function of the hsf family members. Heat Shock Factor.

[55] Rost B. (1999). Twilight zone of protein sequence alignments. Protein Eng..

[56] Yang W.Z., Ko T.P., Corselli L., Johnson R.C., Yuan H.S. (1998). Conversion of a beta-strand to an alpha-helix induced by a single-site mutation observed in the crystal structure of fis mutant pro26ala. Protein Sci..

[57] Bhattacharjee N., Biswas P. (2013). Helical ambivalency induced by point mutations. BMC Struct. Biol..

