# Supplementary material for: Evolutionary Divergence of Duplicated Hsf Genes in Populus
Source: Cells. 2019 May 10;8(5):438. doi: 10.3390/cells8050438 (PMC6563006; doi:10.3390/cells8050438)

Supporting Information

Article title: **Evolutionary divergence of duplicated *Hsf* genes in *Populus***

Authors: Bobin Liu, Jianjun Hu, Jin Zhang

The following Supporting Information is available for this article:

Figure S1 3D structures comparison of paralogous pairs from PtHsf family.

Figure S2 Two paralogous pairs protein sequence alignment analysis.

Table S1 Enrichment analysis of *PtHsfs* co-expressed genes. (included as a separate excel file)

Table S2 List of transcription factor genes co-expressed with *PtHsfs*. (included as a separate excel file)

Table S3 SNP information of *PtHsf* gene family. (included as a separate excel file)

Table S4 protein sequence similarity in Figure 6B. (included as a separate excel file)

Figure S1 3D structures comparison of paralogous pairs from PtHsf family

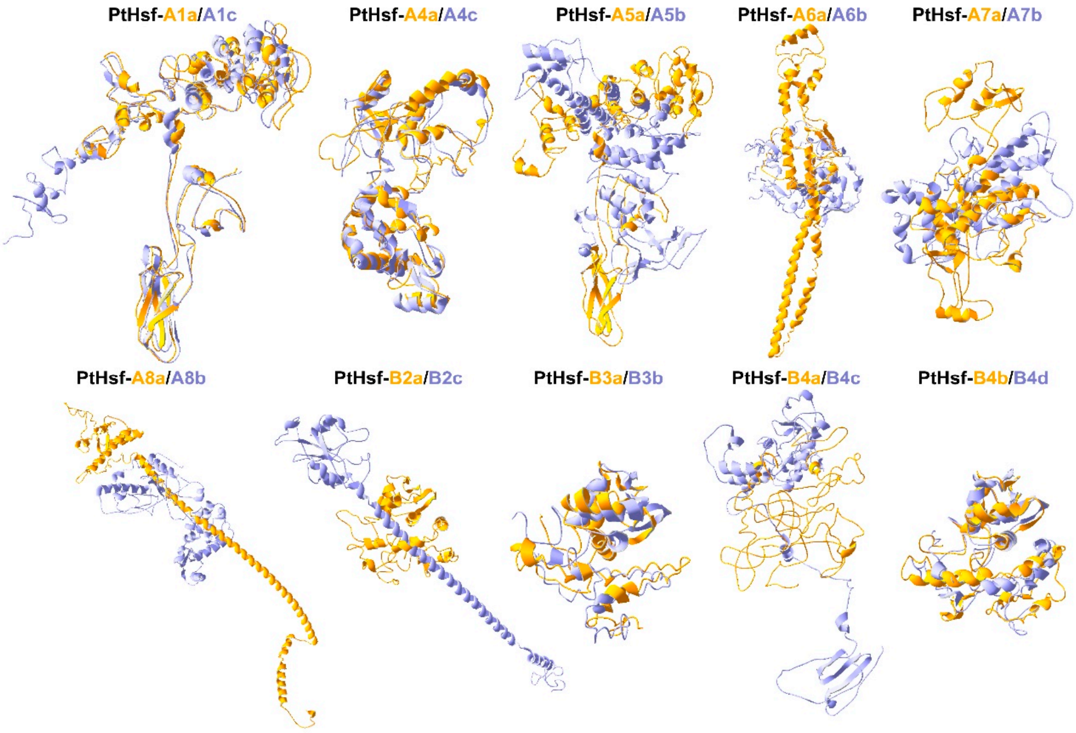

Figure S2 Two paralogous pairs protein sequence alignment analysis

(A) PtHsf-B2a and PtHsf-B2c protein sequence alignment. Blue arrows represent mutation to Pro or Gly mutation.

(B) PtHsf-B8a and PtHsf-B8b protein sequence alignment. Blue arrows represent mutation to Pro or Gly mutation.

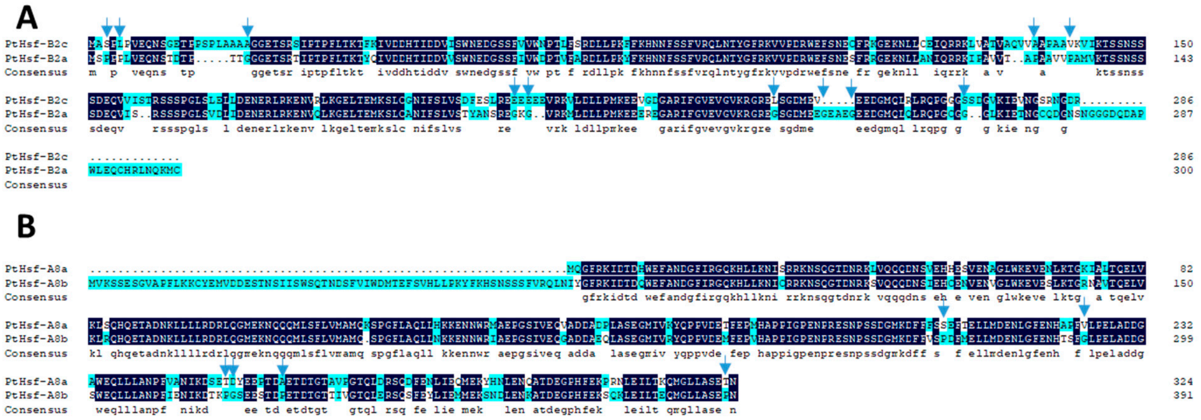

Supplement: Supplementary file 1 [file cells-08-00438-s001.zip › cells-500818-supplementary/supplemental Figures.pdf]
